# Occupational violence and aggression in urgent and critical care in rural health service settings: A systematic review of mixed studies

**DOI:** 10.1111/hsc.14039

**Published:** 2022-09-27

**Authors:** Sharon L. Grant, Stephanie Hartanto, Diane Sivasubramaniam, Kaye Heritage

**Affiliations:** ^1^ Department of Psychological Sciences Swinburne University of Technology Hawthorn Victoria Australia; ^2^ University of Melbourne Parkville Victoria Australia; ^3^ Institute of Health and Wellbeing Federation University Australia Ballarat Victoria Australia

**Keywords:** occupational violence and aggression, rural and remote health, systematic review, urgent or critical care unit

## Abstract

Rural/remote health services are vulnerable to occupational violence and aggression due to factors such as weapon accessibility, poor network coverage and distance to backup. This systematic review investigated (1) the nature of occupational violence and aggression perpetrated in rural/remote health service urgent care settings and (2) the availability and effectiveness of policies/interventions/recommendations that address occupational violence and aggression in this context. We searched Business Source Complete, CINAHL Complete, Health & Society, APAIS Health, Health Collection, PsycINFO, PubMed, Scopus, SocIndex and Web of Science. Included articles (peer‐reviewed, no grey literature and English language) addressed occupational violence and aggression in rural health service urgent care settings. Fifteen articles matched these criteria (total [rural/remote only, where specified] *N* ~ 2555) and were included in the final analysis. The Mixed Methods Appraisal Tool was applied to assess the risk of bias. A data extraction table and narrative synthesis are presented. The most common occupational violence and aggression type was verbal aggression. The primary perpetrator was patients. Risk factors reflected practitioner age, remoteness, sector, staffing, shift type and area of practice. Precipitating factors were alcohol/drugs, dissatisfaction and mental health conditions. Policy content and limitations and education/training programme effectiveness were not addressed. Community collaboration supported occupational violence and aggression prevention/management. Organisational culture should promote reporting, debriefing and post‐incident care for staff well‐being. Work environment and job/task design are priorities for safety, but with possible limitations for traumatised clients. Occupational violence and aggression policies/interventions in rural health settings must be systematically evaluated to inform best practices. *Co‐funded by Swinburne Social Innovation Research Institute Interdisciplinary Seed Funding Scheme and SMART Rural Health Network.*

**What is known about this topic**

- Occupational violence and aggression are estimated to affect 95% of healthcare workers in Australia, placing workers at risk physically and psychologically, with financial implications for organisations, for example, absenteeism, work incapacity claims and costs invested to improve safety.
- Occupational violence and aggression may compromise public health at a broader level by reducing the quality of clinical care.
- Current guidelines for the prevention and management of occupational violence and aggression overlook risk factors in small, rural health services that often have limited staff and resources as well as additional challenges due to remoteness, for example, distance to emergency backup/response.

**What this paper adds**

- This systematic review describes the nature of occupational violence and aggression in rural health services for nurses and healthcare workers generally, including incident characteristics (type, frequency and severity), perpetrator characteristics and risk/precipitating factors.
- Where possible, the review provides an overview of the effectiveness of policies/interventions/recommendations for the prevention and management of occupational violence and aggression in rural health services.
- With all included studies but one conducted in Australia, the findings provide an insight into challenges and gaps in the prevention and management of occupational violence and aggression in rural health services in Australia.

## 1 INTRODUCTION

Occupational violence and aggression describe ‘incidents in which a person is abused, threatened or assaulted in circumstances relating to their work’ (Victorian Department of Health and Human Services – DHHS, 2017). Occupational violence and aggression is a prominent issue across the healthcare sector worldwide (Auditor‐General Victoria, 2015; Kerr et al., 2017; Morphet et al., 2018; Shea et al., 2017) and is estimated to affect 95% of Australian healthcare workers (Spelten et al., 2020). Occupational violence and aggression incidents place workers at risk of physical injury or even death, as well as increasing the risk of burnout and post‐traumatic stress (Kerr et al., 2017; Rees et al., 2018; Spelten et al., 2020). There are also financial implications due to lost days of work, work incapacity claims and costs invested to improve work safety (Hassard et al., 2019; Maguire et al., 2018). Additionally, occupational violence and aggression may reduce the quality of clinical care for a variety of reasons, for example, burnout (Spelten et al., 2020), thus potentially compromising public health at a broader level.

Given the adverse nature of occupational violence and aggression and its implications within healthcare settings, there has been considerable investment in establishing guidelines and protocols to manage it. In Australia, the Victorian Departmental of Health and Human Services recently published the Code Grey Standards, which provide guidelines for ‘an organisation‐level response to actual or potential violent, aggressive, abusive or threatening behaviour, exhibited by patients or visitors, towards others or themselves, which creates a risk to health and safety’ (Victorian DHHS, 2017). However, the standards were described within the context of ‘ideal’ well‐resourced health services, where there are teams of clinicians and security staff, and access to resources, equipment and context‐specific training. Rural health service sites take a range of forms and have varying team sizes, but many are small sites with restricted staffing levels and little or no access to security or legal services (Hills et al., 2019). In addition, rural health services may be vulnerable due to additional challenges including geographical distance to emergency backup/response (Grant & Hartanto, 2019), access of local residents to guns and knives (Lyneham, 2000), poor mobile phone connectivity (Grant & Hartanto, 2019; Thomas et al., 2020), built environments and grounds that are difficult to secure safely (Hills et al., 2019) and reduced privacy of healthcare workers in the community, resulting in a reluctance to pursue incidents (Hills et al., 2019). As such, when occupational violence and aggression incidents occur, it may be difficult for workers to know how best to respond, given these circumstances.

At the time of writing, there were no clear guidelines on how to design locally contextualised occupational violence and aggression practices for implementation within rural health services. Thus, we conducted a systematic review focussing on occupational violence and aggression in rural and remote health services in a high‐risk setting: urgent care centres/emergency departments (Cabilan & Johnston, 2019; Gacki‐Smith et al., 2009; Lau et al., 2004; Nikathil et al., 2017; Spelten et al., 2020). The objectives of the systematic review are to summarise and synthesise evidence relating to occupational violence and aggression perpetrated by patients/visitors in rural health service urgent care facilities, and to inform the management of incidents in rural health service urgent care settings, to support improve health and safety outcomes. Our research questions were as follows:

1. What is the nature of occupational violence and aggression perpetrated by patients/visitors in rural health service urgent care facilities in Australia and internationally?
2. What are the available policies/interventions/recommendations that address occupational violence and aggression perpetrated by patients/visitors in rural health service urgent care facilities in Australia and internationally? What is the evidence for the effectiveness of these policies/interventions/recommendations for rural health sites?

## 2 METHODS

The systematic review followed the preferred reporting items for systematic reviews and meta‐analyses (PRISMA) protocol (Moher et al., 2015). We registered the systematic review protocol with PROSPERO: [https://www.crd.york.ac.uk/PROSPERO/display_record.php?ID=CRD42019131867&ID=CRD42019131867].

### 2.1 Search strategy

We searched the following electronic databases using the default settings: Business Source Complete (EBSCOhost), CINAHL Complete, Health & Society (Informit), APAIS Health (Informit), Health Collection (Informit), PsycINFO, PubMed, Scopus, SocIndex and Web of Science. The search string was (occupation* OR workplace) AND (violence OR aggress* OR ‘behavio* disturbance’ OR ‘code grey’ OR ‘code gray’ OR ‘patient aggression’ OR ‘patient violence’ OR ‘client initiated aggression’) AND (health OR ‘health care’ OR healthcare OR ‘health service’ OR emergency OR hospital OR clinic OR ‘general practice’ OR GP) AND (rural OR remote). The search string included ‘clinic’ and ‘GP’ (general practitioner) as we initially wanted to scope the total number of articles available for rural healthcare services. However, after the initial search, we decided to narrow our review to literature that specifically addresses occupational violence and aggression in rural health service urgent care facilities due to time and budget restrictions.

We did not impose date and language restrictions as we wanted to capture a broad range of local and international literature. For literature in languages other than English, we attempted to find an English language version. The search was conducted on March 3, 2019, and March 4, 2019. Ethical approval was not required as we used published studies in which primary investigators obtained informed consent.

### 2.2 Eligibility criteria

Articles were included if they were peer‐reviewed/refereed literature (including refereed book chapters, qualitative and quantitative studies, mixed‐method studies and review papers) that address occupational violence and aggression in rural health service urgent care settings. Exclusion criteria were as follows: (1) not peer‐reviewed, (2) grey literature, (3) does not address occupational violence and aggression in rural urgent care centre facilities (i.e. occupational violence and aggression occur outside rural urgent care centre facilities, for example, general practitioner clinic, ambulatory transport, outreach health services, mental health or psychiatric facilities and nursing home) unless sampled in aggregate with the population of interest and (4) literature unavailable in English. As we expected limited literature relating to occupational violence and aggression in rural health service urgent care settings, we included aggregated data in rural health service settings inclusive of our population of interest.

### 2.3 Study screening

**FIGURE 1:**
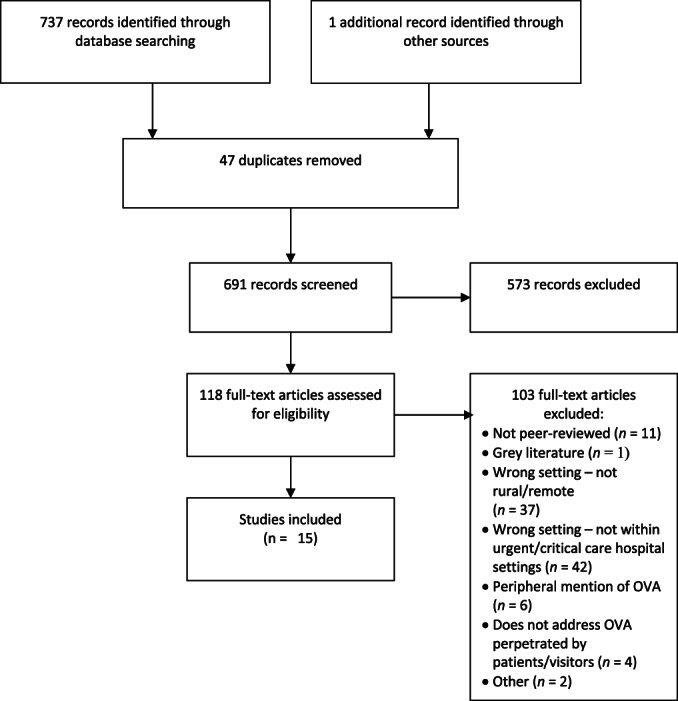
PRISMA flowchart.

The initial search identified 691 records after removing duplicates. Articles were subsequently uploaded to Covidence (www.covidence.org) for screening. First, co‐authors 1 and 2 independently conducted title and abstract screening, whereby literature was sorted into ‘yes’, ‘maybe’ or ‘no’ categories based on the inclusion and exclusion criteria. This resulted in 573 articles being excluded. Next, these co‐authors independently conducted full‐text screening on the 118 ‘yes’ and ‘maybe’ records, recording the reason for exclusion. Disagreements were resolved through discussion or, if required, through consultation with an independent co‐author (3). We excluded 103 articles for not meeting the inclusion criteria and retained 15 articles for inclusion. Figure 1 summarises our process.

### 2.4 Quality assessment

Co‐authors 1 and 2 independently conducted risk of bias (quality) assessments using the Mixed Methods Appraisal Tool (Hong et al., 2018). This tool includes five methodological criteria questions (yes, no and unclear) for each study type (see Mixed Methods Appraisal Tool 2018 user guide; Hong et al., 2018) and is considered an effective and efficient quality appraisal tool for empirical research, with good inter‐rater reliability (Pace et al., 2012). Disagreements were resolved by discussion, with the involvement of an independent co‐author (3) as necessary.

A quality score for each study was calculated based on the percentage of ‘yes’ responses divided by five, with scores ranging from 0% (no criteria met) to 100% (all five criteria met). Quality scores are presented in Table 1. Most studies were of adequate quality: seven studies (46.7%) were high quality, four studies (26.7%) were moderate quality and three studies (20.0%) were low quality. One included article is a literature review and thus was not assessed using the tool. No studies were excluded from the review based on quality scores. Inter‐rater reliability for quality scores was calculated using a two‐way mixed‐model intra‐class correlation coefficient (Koo & Li, 2016). The intra‐class correlation coefficient was 0.57, indicating moderate‐to‐good agreement between assessors.

**TABLE 1:** Data extraction: Included studies

Authors and aim(s)	Methods	Main findings	MMAT score (%)	Certainty score (0–4)
Alexander et al. (2004) assessed the nature and extent of occupational violence in rural health services in Eastern Australia	Sample: Urgent/critical care was included in fields of practice for 114 allied health professionals (72% response rate), 85 general practitioners [62% response rate] and 762 nurses (62% response rate). Respondents were similar to non‐respondents in age and gender but nurse respondents tended to have more tenure than nurse non‐respondents Measures: Cross‐sectional postal survey of occupational violence from patients, relatives and colleagues, workplace hazards, barriers to work safety and demographics Statistical tests: Cross‐tabulations with chi‐square	68% of nurses, 47% of allied health professionals and 48% of general practitioners experienced occupational violence The most distressing source of occupational violence was patients The most frequent form of occupational violence was verbal abuse, followed by threatening behaviour, physical violence and obscene behaviour Service issues (allied health professionals), drug and alcohol issues (general practitioners) and psychiatric issues (nurses) were the most common precipitating factors Demographics and occupational violence were unrelated Qualitative comments indicated that occupational violence affects stress levels, recruitment/ retention and the provision of services Telephoning police was a major defence strategy for allied health professionals and nurses. Nurses responded by revising policy, security and aggression management in the workplace; general practitioners changed for whom and where they offered health services	80	Sample: 2 RQ1: 4 RQ2: 2 Average: 2.67
Beattie, Griffiths, et al. (2018a) explored the effect of previous client trauma on workplace violence as part of a larger descriptive study on guidelines for managing workplace violence	Sample: 99 volunteer participants, from Occupational Health and Safety, emergency departments and home visiting services and occupying various roles, in regional (57.6%), city (39.4%) or specialist hospitals (3%) Measures: Individual and group interviews. Questions not published Statistical tests: N/A (qualitative)	Participants noted the challenges of engaging clients with altered cognitive states. Care provision (e.g., directing/restricting clients) could be perceived as a threat, resulting in workplace violence Participants suggested trauma‐informed care to prevent and manage workplace violence but not all participants/health services acknowledged educational, policy and procedural responses	20	Sample: 1 RQ1: 2 RQ2: 2 Average: 1.67
Beattie, Innes, et al. (2018b) examined neurobiological responses (fight, flight and freeze) that could be occurring when healthcare workers are exposed to workplace violence to inform future training and self‐care strategies for staff well‐being. Again, this study was part of a larger descriptive study on guidelines for managing workplace violence	As per the previous study	Workplace violence tolerance was high, especially in ED, aged care and mental health The inability to plan care was cited as a major issue, especially in home care environments and staff relied on gut instinct rather than formal risk assessment Physical and psychological barriers (e.g., environmental design and signage) discourage social engagement (communication, connection and support) among workers and consumers which can negatively impact safety Trauma‐informed care training was recommended to address cumulative trauma responses among workers Staff often declined support from Employee Assistance Programs. Debriefing and management post‐incident were inconsistently offered and frequently delivered by staff with no formal training. Supporting others, functional teamwork, support from the ground up and top down, good communication strategies/skills and self‐care were described as important for resilience Current training focuses on [rare] extreme physical violence, for example, physical restraint. A training package that includes staff safety, the effects of cumulative trauma and trauma‐informed care for all areas of the health service was seen as important	20	Sample: 1 RQ1: 2 RQ2: 2 Average: 1.67
Brock et al. (2009) examined how physicians utilise the secure room/security guard system for patients who are potentially violent, aggressive or suicidal, and the disposition of these patients	Sample: Three‐year, retrospective patient chart audit at a rural hospital. The study did not involve the active participation of individuals Measures: The authors obtained a log from the security firm the hospital contracted, and reviewed patient charts to determine the number of patients who were admitted to the security room, reason for admission and characteristics and disposition of these patients Statistical tests: Descriptive statistics	35,115 patients were admitted to the emergency department over the survey period. The secure room/security guard system was utilised 39 separate times. The average use of the secure room/security guard system was slightly more than once per month, or at a rate of 1.1 uses/1000 emergency department visits The most common reason for admission was the perceived risk of suicide or self‐harm (59%). Concern for staff safety was cited in 10% of the admissions	100	Sample: 0 (N/A) RQ1: 2 RQ2: 2 Average: 1
Farrell et al. (2014) explored experiences of patient and visitor assault and the relationship between assault and ‘protective’ employer factors	Sample: 1495 nurses and midwives from 5000 registered with Nurses Board of Victoria Survey (30% response rate; 66% metropolitan) Measures: Participants were asked to report on their experiences of patient and visitor assault in the past 4 working weeks. They were then asked to (a) rate the importance of 26 ‘protective’ factors to help prevent and manage assault and (b) indicate the presence of factors in their work setting. Statistical tests: Descriptive statistics (percentages and frequencies) and binary regression analysis (presence of protective factors as a predictor of patient and visitor assault) with control of potential covariates: nursing division, work location, work type, employment, duty roster and clinical setting	36% of participants reported patient visitor assault in the past 4 working weeks and 46% reported three or more incidents. Verbal abuse (90%), physical abuse (45%) and threat of harm (27%) Patients were approximately 2.5 more times assaultive than visitors Men aged over 50 years accounted for 54% of patient visitor assault incidents Protective factors were high standard facilities, personal protective equipment (mobile phones and personal duress alarms), sufficient staffing levels and effective enforcement of policies by management (policy details not provided) Risk factors were working in public settings; accident and emergency, aged care and mental health settings; working on a rotating roster and working on night duty Aggression management training was neither a protective factor nor a risk factor for patient visitor assault; the authors speculated that training could be *‘inadequate’*	60	Sample: 1 RQ1: 4 RQ2: 3 Average: 2.67
Fisher et al. (1996) examined violence directed towards remote area nurses as a problem in remote communities	Sample: 237 remote area nurses who were members of Council of Remote Area Nurses Australia (41.35% response rate for questionnaires). The sample for interviews consisted of four remote area nurses Measures: Questionnaire: experiences with violence and related issues: verbal aggression and obscene behaviour, property damage, telephone threats, stalking, physical violence, sexual harassment and sexual abuse (all accompanied by plain language definitions). One open‐ended question asked participants to describe a violent incident that had affected them within the previous 6 months Unstructured teleconference interviews. Statistical tests: Frequency distributions and cross‐tabulations	Verbal aggression and obscene behaviour (82.1%), property damage (46.7%) and physical violence (48.1%) were the most commonly experienced Remote area nurses aged 20–29 were more likely to experience sexual harassment on duty and on call More violence occurred in smaller communities, both on duty and on call; respondents on call 24 h were more likely to experience all types of violence Respondents without a security escort were more likely to experience verbal aggression and obscene behaviour on‐call, physical violence on duty and on call and sexual harassment on and off duty Those living in independent accommodation were the least likely to experience violence Perpetrators tended to be male, Aboriginal and either a client or his family Violent incidents tended to occur mostly at night The location of violence was mostly frequently the clinic/hospital Incidents were associated with a range of negative emotions yet there were significant numbers who perceived the frequency and severity of incidents as low, except with physical violence Broader social context, for example, alcohol (all participants), dissatisfaction with the service provided, unreasonable/illegal requests, mental illness, grieving/sadness and rioting, were identified as contributing factor The majority of participants did not feel adequately prepared for their work (46.9% had no orientation prior to commencing their current position and 30.8% had no orientation or employer‐based education since commencement). Only 53.6% received cross‐cultural information; 24.3% received personal safety information There was little or no support post‐incident reporting apart from temporary leave; 32.8% no longer felt confident reporting future incidents. Only 52.8% always reported incidents	0	Sample: 3 RQ1: 4 RQ2: 1 Average: 2.67
Hegney et al. (2003) examined how nurses perceive their work and their working conditions	Sample: Stratified (public, private and aged care), random sample of 2800 active members of the Queensland Nurse's Union. The final sample consisted of 441 (47%) aged care, 497 (56%) public and 498 (56%) private Measures: Descriptive, self‐report postal survey containing six workplace violence questions: ‘In the last 3 months at your workplace have you been attacked, bullied, threatened, harassed or otherwise abused?’ (if yes, indicate the source and number of such incidents occurring in the last 3 months). ‘Does your workplace a policy for dealing with aggressive behaviour of other staff?’. (if ‘yes’ indicate if the policy was ‘adequate’ in dealing with this issue; response scale as previous). ‘Does your workplace have a policy for dealing with aggressive behaviour of non‐staff (patients/clients/visitors)?’ (if ‘yes’ indicate if the policy was ‘adequate’ in dealing with this issue; response scale as previous) Statistical tests: Weighted percentages, chi‐square analysis	Within the previous 3 months, workplace violence was experienced by 74% of aged care workers, 63% of public sector workers and 48% of private sector workers Aged care only: workplace violence prevalence was significantly higher for women than men. Registered nurses Level 1 experienced more workplace violence from visitors and relatives than other designations 74% of aged care, 64% of public and 55% of private respondents were aware of workplace violence policies. Private respondents perceived workplace violence policies as significantly more adequate compared to aged care and public respondents Public sector only: Workplace violence policies were significantly less prevalent in remote areas (52%) than in capital cities (66%) and large rural centres (71%). Men perceived policies as less effective than women. Perceived inadequacy of policies increased with the level of experience	80	Sample: 1 RQ1: 1 RQ2: 3 Average: 1.67
Hills (2008) examined the relationship among rural general nurse's experiences of patient aggression, participation in aggression management training and self‐efficacy in dealing with patient aggression. The author sought to broadly address the paucity of nursing research on patient aggression in Australian rural general hospital settings	Sample: Simple random sample of 650 general hospital nurses chosen from a population of 901 nurses working in permanent, full‐time or part‐time positions in general hospital and small, rural health services incorporating state‐funded acute care beds and Commonwealth‐funded aged care beds. Participants were located in central west New South Wales, Australia. Response rate was 48.3% (*N* = 300) The proportion of female nurses and the age of the sample was slightly higher than in the population of nurses in New South Wales at the time of the study. Measures: demographic questions, a single item related to the frequency of participation in aggression management training or updates in the previous 5 years; items type of patient aggression experienced in the previous 3 months and the resulting number of days taken off work; and the 5‐item efficacy in Dealing with Challenging Behaviours Scale (Hastings & Brown, 2002) Statistical tests: Factor and reliability analysis of Dealing with Challenging Behaviours Scale and correlational analysis of ordinal and interval variables	The median number of times participants had received aggression management training in the previous 5 years was once; 39.5% had not participated in training, 36% had received training once, 17.2% twice and 6.9% three or more times Excluding managers and specialists/consultants, 76% of participants had experienced patient aggression in the previous 3 months. Registered nurses and midwives were most affected (83.4%) The most common form of aggression was verbal abuse, followed by verbal threats and intimidation, physical threats and intimidation and physical violence 96.7% had taken no days off work, 2.4% one or 2 days and two participants had taken either 10 days or 60 days off work 70% of participants reported self‐efficacy in the low‐to‐medium range There was a moderate association between verbal threats or intimidation and physical threats or intimidation, suggesting co‐occurrence. Participation in aggression management training showed a weak, negative association with verbal abuse experienced, and a weak, positive association with perceived self‐efficacy	60	Sample: 3 RQ1: 3 RQ2: 3 Average: 3
Jackson and Ashley (2005) sought to determine the prevalence of occupational physical and psychological violence that health staff experience in Jamaica and to identify factors associated with experiences of violence	Sample: 832 health staff from urban (*n* = 670) and rural (*n* = 157) health services including private and public hospitals, and primary healthcare facilities. The sample included nurses, administrative or clerical staff, support staff, ancillary staff, physicians, midwives, ambulance officers, allied health professionals, etc Measures: Cross‐sectional design, utilising a standardised questionnaire (administered by verbal interview) exploring health workers' experiences of physical and psychological violence in the preceding year Statistical tests: Descriptive statistics, univariate logistic regression and multivariate logistic regression	7% of health staff reported physical violence in the preceding year, more commonly male staff (9.7%) than female staff (7.1%); staff aged 44 years and younger were more likely to experience physical violence than older staff members and, within the hospital setting, the largest proportions of violent incidents occurred within general medicine (30%), psychiatric wards (17%), administration units (13%) and emergency units (11%) Psychological violence was more common than physical violence; 33%–44% of the staff of various age groups reported verbal abuse in the preceding year. Staff aged 34 years and below reported higher levels of verbal abuse compared to older staff Patients were the primary perpetrators of both physical and psychological violence The risk of physical violence was reduced among staff who were 55 years and older, worked at night (6:00 p.m. to 7:00 a.m.), or worked with mostly mentally disabled, geriatric or human immunodeficiency virus/acquired immune deficiency syndrome patients; physicians and nurses were at an increased risk of physical violence; and staff who work mostly with psychiatric patients were at an increased risk of physical violence	80	Sample: 1 RQ1: 4 RQ2: 0 Average: 1.67
Lyneham (2000) sought to define violence from the perspective of New South Wales emergency nurses’ perspectives, identify their perception of violence and examine personal and managerial responses to violent incidents	Sample: Stage 1 (qualitative interviews): 9 New South Wales nurses in rural (*n* = 3), remote (*n* = 3) and metropolitan (*n* = 3) emergency departments. Stage 2 (questionnaires): 266 New South Wales nurses from across rural (*n* = 65), remote (*n* = 21) and metropolitan (*n* = 180) emergency departments Measures: Interviews addressed understanding and definition of violence in the emergency department; personal experience of violence; effects of violence on the participant; role of management; type of violence experienced; location of violence; use of weapons; source of violence; police and security staff response time; precipitating factors; administration responses and training to deal with violent situations. The questionnaire addressed: types of security devices used in the workplace; working order of the security devices; types and frequency of violence; level of fear; frequency of incidents involving weapons; perpetrators of violence; response time of hospital security staff and police; precipitating factors; percentage of incidents reported in writing; satisfaction with the administrative response; time to get support for the most significant incident; policies on violence and their adequacy; and training adequacy Statistical tests: Descriptive statistics	Quantitative findings: Alcohol (94%), waiting time (85%) and drugs (83%) were the most common precipitating factors of violence in rural emergency departments. Alcohol (95%), drugs (62%) and socioeconomic factors (62%) were the most three common precipitating factors of violence in remote emergency departments 100% of rural and remote emergency nurses reported verbal abuse (figures for times per year, per month, weekly and daily are also provided for all forms of violence—see article) The following figures correspond to rural and remote emergency nurses respectively: 97% and 99% reported verbal abuse by phone 93% and 57% reported physical intimidation or assault. 100% and 38% reported threats 74% and 78% were sometimes fearful at work; 22% and 14% were often fearful at work 42% and 8% reported feeling a little dissatisfied or very dissatisfied with administrative responses to violence 54% and 48% reported violence‐related policies 34% and 23% had never been offered training for violent incidents 15% and 24% of violent incidents were usually reported. 14% and 19% were never reported. 53 and 4 violent incidents involved the use of weapons such as a gun, knife, hospital equipment and other weapons Patients and their relatives and/or friends were the most common perpetrators Lack of reporting was attributed to the belief that nothing would be done by the administration, or the perception of violence as part of the job. Several nurses indicated that the administration can be punitive and blaming of staff For hospitals with security staff, approximately 52% of calls were answered within 10 min. Police responded within 30 min 84% of the time Qualitative findings: Despondency and feelings of helplessness and hopelessness were apparent for emergency nurses Respondents expressed frustration with the legal system and insufficient penalties Some respondents commented on workplace design flaws, for example, no escape routes, and security doors cannot prevent violence from patients and/or visitors inside	40	Sample: 4 RQ1: 4 RQ2: 3 Average: 3.67
Luck et al. (2009) sought to observe routine strategies that emergency nurses utilise when interacting with patients to avert, reduce or prevent violence, and gain insights into violence prevention strategies routinely used by rural emergency nurses	Sample: 20 emergency nurses from a regional Australian emergency department servicing a large rural and remote multi‐cultural community Measures: Instrumental case studies, informal field interviews, semi‐structured interviews, researcher journaling and both structured and unstructured participant observation Statistical tests: Not applicable	Observational and narrative data revealed five broad attributes that emergency nurses demonstrated in practice when interacting with patients and visitors to avert or reduce violence: Being safe—prioritising safety, managing the physical environment and utilising high‐quality, professional interpersonal skills Being available—acknowledging that the emergency department is likely to be a stressful environment for patients and visitors, and using communication skills to convey empathy and letting the patient/visitor know that staff are available for support Being respectful—conveying respect to patients and visitors by building rapport, communicating in a calm demeanour, using non‐threatening body language, working harmoniously with patients and visitors and setting respectful boundaries Being supportive—ensuring that patients and visitors feel supported by making them feel comfortable and keeping them informed about various emergency processes, and using unambiguous and jargon‐free communication Being responsive—responding to patient's emotional and physical needs, including needs that the patient has not explicitly stated	80	Sample: 4 RQ1: 0 RQ2: 2 Average: 2
Magin et al. (2010) explored the occupational violence experiences of rural general practitioner registrars	Sample: 15 New South Wales general practitioner registrars who had completed or were completing mandatory training in rural areas. *General practitioners were included here due to the hospital context* Measures: semi‐structured interviews Statistical tests: Not applicable	Female general practitioner registrars reported significant concerns relating to occupational violence and aggression during their rural term, while male general practitioner registrars did not Perception of risks tended to be heightened after hours due to the isolation and lower staffing levels in rural hospitals and police forces. Registrars reported that their security concerns were not always taken seriously by supervisors Experiences of occupational violence and aggression and perceived risk are significant stressors for some registrars and may precipitate attrition from training programmes	100	Sample: 3 RQ1: 3 RQ2: 0 Average: 2
Mayhew and Chappell (2003) sought to establish baseline estimates of occupational violence among the public health workforce in one Australian state. *As our systematic review focuses on occupational violence and aggression in rural/remote urgent/critical care settings that are perpetrated by patients and visitors*, *we focus our report on the variations in violence patterns between rural and urban areas. The remaining data reported in this study mostly aggregated rural and urban responses*, *as well as internal and external occupational violence*, *which made it difficult for us to separately examine rural/remote‐specific emergency department/urgent and critical care data that focuses on violence perpetrated by patients and/or visitors*	Sample: 400 public health workers including 40 allied health workers, 40 ambulance officers, 80 ancillary staff, 40 medical officers and 200 nurses from 45 different hospitals, 14 ambulance stations and several community health services. Includes four geographical areas, including two urban (*n* = 200) and two rural areas (*n* = 200) Measures: Semi‐structured interviews that included quantitative and qualitative questions addressing a number of violent events over the preceding 12 months, witnessing of violence towards other staff members, perceived high‐risk places, perpetrators of violence, violence management strategies, recommendations for violence prevention, other employment‐linked violence issues, contexts or scenarios and severity of violent events. Additionally, the abbreviated 12‐item General Health Questionnaire (GHQ‐12) was included to measure emotional distress associated with occupational violence experiences Statistical tests: Descriptive statistics, analysis of variance and regression analyses	Participants reported experiencing 585 separate violent events over the preceding 12 months including verbal abuse, threats, assaults, bullying and other events Results indicated that higher‐risk people and situations may be concentrated in certain geographical regions Qualitative responses suggested difficulties due to not having a medical officer on site when patients with a wide range of conditions presented at the emergency department, and increased risk due to limited staff after hours Emergency departments, intensive care units, critical care units, rural healthcare settings and remote rural sites at night were among the sites identified as having a high risk for violence based on the collated evidence from 400 health workers. Clients were the perpetrators in over three‐quarters of all violent events, while relatives or visitors were the perpetrators in nearly one‐third of threat and verbal abuse events Client‐/patient‐initiated violence was reported to be most common among those who suffer from injury, illness, brain injury, dementia or a semi‐comatose state, mental health problems, those affected by drugs and/or alcohol and those recovering from anaesthesia Relative‐/visitor‐initiated violence was more commonly perpetrated by people from lower socio‐economic backgrounds, although some reported perpetrators worked in skilled jobs Perpetrators were identified as male in 49% of the events, female in 23% and both male and female in 5% (gender was not reported in 23% of the events) The authors estimated that only between 8% and 12% of all occupational violence and aggression events are formally reported and recorded on official databases	100	Sample: 2 RQ1: 3 RQ2: 3 Average: 2.67
Opie et al. (2011) summarised the literature on occupational stress among remote area nurses, focusing on workplace violence and strategies to reduce and minimise its detrimental effects	Literature review	Workplace violence was identified as one of the main stressors facing remote area nurses, contributing to high levels of turnover The risk of occupational violence towards remote area nurses cannot be eradicated, as their role involves interacting with people in often highly charged and stressful conditions; however, there are strategies to reduce its impact, including: increasing public awareness and gaining community support through the development of campaigns with a variety of partners including the police, media and victim support centres; creating and maintaining an organisational culture that supports anti‐violence programmes; improving the physical work environment and equipment design; job and task design, for example, ensuring nurses do not work alone; staff training and education on promoting positive relationships with community members, aggression management, de‐escalation strategies and identifying behavioural precursors to violence; improving emergency situation response, for example, educating staff on response strategies when violence occurs; and encouraging staff to report occupational violence and aggression incidents and ensuring staff are supported following incidents	N/A	Sample: 0 (N/A) RQ1: 0 RQ2: 2 Average: 0.67
Ross‐Adjie et al. (2007) Identifies stress‐evoking incidents that emergency department nurses perceive as most significant, investigates whether demographic characteristics influence perceptions and discusses current debriefing practices	Sample: 156 nurses from metropolitan (*n* = 140) and rural (*n* = 7) emergency departments in Western Australia. Nine nurses did not report their location Measures: Demographic information, professional characteristics and various stressors that nurses were asked to rank from ‘1’ (most stressful) to ‘15’ (least stressful). A qualitative question explored debriefing practices following stressful incidents Statistical tests: Descriptive statistics, Kruskal–Wallis test, and Mann–Whitney *U*‐test	‘Violence against staff’ was ranked as the top stressor 41% of respondents had sought debriefing following a stress‐evoking incident, approx. 80% were offered debriefing and 59% reported that routine debriefing was not offered Respondents reported that the shift coordinator or most senior nurse on duty often chaired debriefing sessions, but several felt that it should be done independently, to maintain confidentiality. Some reported that debriefing was not helpful due to the debriefer being ill‐equipped Even if debriefing was offered, staff were sometimes unable to attend, with no relief from duties Some respondents identified the need for ongoing rather than one‐off, debriefing	60	Sample: 1 RQ1: 1 RQ2: 3 Average: 1.67

Co‐authors 1 and 2 independently assessed the certainty of the body of evidence, as related to the objectives of the review and the research questions (see Table 1). We assessed the certainty of evidence as high (4), moderate (3), low (2), very low (1) or uncertain/not applicable (0) with regard to the following criteria for upgrading the certainty of evidence: (a) Relevance of the sample: (1) data are aggregated but include rural health services, healthcare workers working in rural health services or healthcare workers working in emergency/urgent care in rural health services (very low); (2) data are specific to various healthcare workers in rural health services (low); (3) data are specific to a particular subgroup of healthcare workers in rural health services (moderate) or (4) data are specific to emergency/urgent and critical care healthcare workers in rural health services (high); (b) (i) Relevance to RQ1: (1) describes one of the following criteria: occupational violence and aggression type, frequency, severity, source/perpetrator characteristics and risk factors (very low); (2) describes two of the previous criteria (low); (3) describes three of the previous criteria (moderate) or (4) describes at least four of the previous criteria (high) and/or (ii) Relevance to RQ2: (1) broadly discusses policy or interventions (very low); (2) makes broad recommendations for policy or interventions, based on evidence (low); (3) discusses the effectiveness of specific policies or interventions (moderate) or (4) discusses the effectiveness of specific policies and interventions (high). We averaged scores across criteria (a) and (b) to generate a total score (0–4). Scores ranged from 0.67 to 3.67. Inter‐rater reliability (intra‐class correlation coefficient) for certainty was 0.98 indicating excellent agreement.

### 2.5 Data extraction

For each article, we extracted the objectives, methods and findings (see Table 1).

Articles were divided randomly among co‐authors 1 and 2 and samples of each co‐authors' extraction (approximately 30%) were checked for consistency in the early stages.

A narrative synthesis, structured around the nature and prevalence of occupational violence and aggression incidents, policies/interventions/recommendations to manage incidents and the effectiveness of these policies/interventions/recommendations, is presented below. Quantitative synthesis was not possible as the studies were not sufficiently homogeneous due to varying samples, research methods and data analytic approaches. In addition, some studies provided disaggregated data for the population of interest (rural health services and urgent and critical care/emergency department settings), while others did not. Samples are reiterated throughout the discussion to give context to the findings and highlight limitations of available literature. In addition, there were no data relating to (1) the prevalence of occupational violence and aggression perpetrated by patients/visitors in rural urgent care health service settings; (2) the effectiveness of occupational violence and aggression policies/interventions/recommendations in rural health settings and (3) health and safety outcomes, such as the number of occupational violence and aggression incidents effectively managed (i.e. no harm or injury) or the number of incidents resulting in harm or injury. Despite varying terminology across studies, we use the term ‘occupational violence and aggression’ throughout to refer to *any work situation in which a healthcare worker is abused*, *threatened or assaulted by non‐staff members*.

## 3 RESULTS

All included studies but one (Jackson & Ashley, 2005) were conducted in Australia. Seven focused on nurses and midwives (Farrell et al., 2014; Fisher et al., 1996; Hegney et al., 2003; Hills, 2008; Lyneham, 2000; Opie et al., 2011; Ross‐Adjie et al., 2007) and one study on general practitioner registrars (Magin et al., 2010). The remaining studies included a range of workers (Alexander et al., 2004; Beattie, Griffiths, et al., 2018a; Beattie, Innes, et al., 2018b; Brock et al., 2009; Jackson & Ashley, 2005; Mayhew & Chappell, 2003).

Ten studies addressed the first research question; however, most of these studies aggregated data across different health settings rather than reporting data specific to rural/remote urgent care health service settings; this is noted where applicable in the Discussion. Key themes were incident characteristics, perpetrator characteristics and risk factors. With regard to research question 2, two studies (Hegney et al., 2003; Lyneham, 2000) addressed occupational violence and aggression polices for patients/visitors (see also Farrell et al., 2014 in ‘Interventions and Recommendations’ section of our Discussion) and almost all studies included interventions and/or recommendations that addressed four themes: ‘pushing the training agenda’, ‘lockdown and surveillance’, ‘immediate and ongoing staff support and safety’ and ‘emergency service presence and linkages’. An integrated discussion of findings is provided below.

## 4 DISCUSSION

### 4.1 RQ. 1: What is the nature of occupational violence and aggression perpetrated by patients/visitors in rural health service urgent care facilities?

#### 4.1.1 Incident characteristics: Type, frequency and severity

##### Nurses

Fisher et al. (1996) found that verbal aggression and obscene behaviour, property damage and physical violence were the most common forms of occupational violence and aggression among Australian rural area nurses. With the exception of physical violence, severity was perceived as low, possibly reflecting acculturation to cultural violence in the community.

In Lyneham's (2000) study of Australian rural and remote emergency nurses, verbal abuse and threats were the most prevalent form of occupational violence and aggression, followed by verbal abuse by phone and physical intimidation or assault respectively. There were more incidents involving weapons in rural health services than remote ones.

With regard to severity, Ross‐Adjie et al. (2007) found that ‘violence against staff’ was ranked as the top stressor among Australian emergency department nurses. However, the type and frequency of occupational violence and aggression were not measured in this study, and data were aggregated for rural and metropolitan respondents.

Hills (2008) found that the most common form of occupational violence and aggression for Australian rural nurses was verbal abuse, followed by verbal threats and intimidation, physical threats and intimidation and physical violence. Drill‐down data for areas of practice were not provided. Excluding managers and specialists/consultants, more than three‐quarters of participants had experienced patient aggression in the previous 3 months, with registered nurses and midwives most impacted. There was a moderate association between ‘verbal threats or intimidation’ and ‘physical threats or intimidation’, suggesting co‐occurrence.

Farrell et al.'s (2014) study of Australian nurses and midwives found that the main types of occupational violence and aggression experienced were verbal abuse, physical abuse and the threat of harm in that order; almost 40% of participants had experienced occupational violence and aggression in the past 4 weeks, with half reporting three or more incidents. Rural and regional nurses and midwives were overrepresented in this sample and data were aggregated across all geographical locations.

##### Health services

A study of occupational violence and aggression in Jamaican urban and rural health services (Jackson & Ashley, 2005) found that psychological violence was considerably more common than physical violence over a 1‐year period; however, urban and rural data were aggregated.

Mayhew and Chappell (2003) examined occupational violence and aggression in various occupational groups in urban and rural health services in Australia. Their findings indicated that threats, verbal abuse and assaults from those affected by drugs and/or alcohol may be concentrated in certain (rural) geographical regions.

Alexander et al. (2004) examined occupational violence and aggression among allied health professionals, general practitioners and nurses in Australian rural health services. Verbal abuse was the most frequent form of occupational violence and aggression followed by threatening behaviour, physical violence and obscene behaviour. Overall, incidents were more prevalent among nurses than allied health professionals and general practitioners, with the latter two groups reporting similar levels. Nurses more commonly reported all forms of violence, except telephone threats, which were reported just as commonly by general practitioners. Urgent care was among the fields of practice in this study, but drill‐down data were not available.

A study (Brock et al., 2009) focusing on a secure room in an Australian rural health service reported average use was about once per month, or at a rate of 1.1 uses/1000 emergency department visits. Concern for staff safety was cited in 10% of the admissions.

Beattie et al. (Beattie, Griffiths, et al., 2018a; Beattie, Innes, et al., 2018b) conducted a study in Australian metropolitan and regional (remote) health services focussing on healthcare workers who were responsible for occupational violence and aggression prevention and management or who had experienced incidents. They found that there was a normalisation of occupational violence and aggression due to its frequency, especially in emergency departments, aged care and mental health. Metropolitan and regional data were reported in aggregate.

Together, these studies suggest that verbal aggression (abuse, threats and intimidation) is the most common type of occupational violence and aggression. Other types include obscene behaviour, property damage, verbal abuse by phone and physical aggression (abuse, assaults and intimidation). Given that all but one study discussed in this section focuses on the Australian context, we elaborate the discussion of our findings accordingly.

Our findings in the rural healthcare setting are consistent with studies of Australian nurses and midwives in a range of geographical settings, which suggest these healthcare workers are regularly subjected to unacceptably high levels of occupational violence and aggression (Pfich & Roche, 2020). The impact of occupational violence and aggression on healthcare workers has implications for turnover, which is particularly concerning for rural health services given existing workforce shortages in rural areas (Parliament of Australia, 2022). With access to specialised security staff lacking (Hills et al., 2019), possible interventions might include building de‐escalation skills among rural healthcare workers, including upskilling of workers in behavioural risk assessment and trauma‐informed care. Graded interventions for dealing with behaviours of concern are also needed to reinforce a zero‐tolerance culture in rural health services and their communities.

#### 4.1.2 Perpetrator characteristics

For Australian remote area nurses, perpetrators tended to be male, Aboriginal clients or their family members (Fisher et al., 1996). For Australian rural and remote emergency department nurses, patients and their friends/relatives were the most common perpetrators (Lyneham, 2000). In Mayhew and Chappell's (2003) study (Australian rural and urban data aggregated), clients perpetrated over three‐quarters of violent events, while relatives/visitors perpetrated nearly one‐third of threat and verbal abuse events. Relative/visitor‐initiated violence was more commonly perpetrated by people of lower socio‐economic backgrounds, although some perpetrators worked in skilled jobs. Around half of the perpetrators were male. In Alexander et al.' (2004) study of Australian rural health services, respondents (various areas of practice) were unanimous that patients were the most distressing source of occupational violence and aggression. Farrell et al. (2014) found that patients were 2.5 times more assaultive than visitors, with men over 50 years accounting for more than half of the occupational violence and aggression incidents (data were aggregated across geographical locations in this Australian study). In the only international study included (Jackson & Ashley, 2005), patients were reported to be the primary perpetrators of both physical and psychological violence (urban and rural data were aggregated), consistent with Australian findings. Although patients are the primary perpetrators of occupational violence and aggression across all included studies, visitors are also consistently reported as perpetrators. Internal alert systems such as flagging the files of individuals (clients and visitors) with a history of occupational violence and aggression and sharing of risk profiles across local health and community services could support planned, coordinated responses in rural healthcare settings.

#### 4.1.3 Risk factors

In Fisher et al.' (1996) study of Australian remote area nurses, risk factors for all forms of occupational violence and aggression were working in a smaller community and working on‐call. Age (20–29) was a risk factor for sexual harassment only. Precipitating factors were alcohol, service dissatisfaction, unreasonable/illegal requests, mental illness and grieving/sadness. Incidents occurred mostly at night and in the clinic/health service. Security escorts and independent accommodation were protective factors but may not be available in all rural healthcare settings (Hills et al., 2019).

Similarly, Lyneham (2000) found that alcohol, waiting time and drugs were the most three common precipitating factors of violence in Australian rural emergency departments. In contrast, alcohol, drugs and socioeconomic factors were the most three common precipitating factors in remote emergency departments in this study, suggesting that risk factors vary by local context. In Mayhew and Chappell's (2003) study (Australian rural and urban data aggregated), patient‐initiated violence was most common among those who suffer from injury, illness, brain injury, dementia, mental health problems, substance abuse and anaesthesia aftereffects. Qualitative responses of several interviewees from rural health services in Mayhew and Chappell's study suggested not having a medical officer on site at the emergency department, and limited staff after hours were additional risks. Emergency departments, intensive care units, urgent and critical care, rural healthcare settings and remote/rural sites at night were among the settings identified as a ‘high risk’ for violence based on the collated evidence from 400 health workers.

In a Jamaican study (Jackson & Ashley, 2005), staff aged 34 years and below reported higher levels of verbal abuse compared to older staff, consistent with Australian findings that younger healthcare workers face a higher risk. Physical violence was slightly more common among staff aged 44 years and below, male staff and within general medicine, psychiatric wards, administration units and emergency units, respectively. The risk of physical violence was reduced among staff who were older, worked at night suggesting possible differences in staffing levels compared with the Australian context (Magin et al., 2010) or worked mostly with mentally disabled, geriatric or human immunodeficiency virus/acquired immune deficiency syndrome patients. Physicians and nurses were at an increased risk of experiencing physical violence overall, and staff who worked mostly with psychiatric patients were at increased risk of physical violence compared with those who worked with other patients (urban and rural data were aggregated). Similarly, the most frequent precipitating factor for occupational violence and aggression among nurses in Alexander et al.'s (2004) study of Australian rural health services was patient psychiatric issues. Service issues (allied health professionals) and drug and alcohol issues (general practitioners) were also reported as precipitating factors. A study in Australian metropolitan and regional (remote) health services (Beattie, Griffiths, et al., 2018a; Beattie, Innes, et al., 2018b) noted the challenges of engaging clients with altered cognitive states: stress and frustration and previous trauma can compromise clients' assessment of risk and safety, resulting in inappropriate reactivity to workers providing care. Metropolitan and remote data were aggregated in this study.

Magin et al.' (2010) study of Australian rural general practitioner registrars found that perceived risk was higher after hours than during business hours due to isolation, lower staffing levels and reduced police presence. Occupational violence and aggression, including perceived risks, was a significant source of stress particularly for female, young or less experienced registrars.

In Farrell et al.' (2014) study of Australian nurses and midwives, risk factors for occupational violence and aggression were working in public healthcare settings; accident and emergency, aged care and mental health settings; and a rotating roster and night duty. Rural and regional nurses and midwives were overrepresented in this sample, and data were aggregated across all geographical locations included.

The above studies indicate that risk factors for occupational violence and aggression include age of practitioner (younger), rurality/remoteness, sector (public), staffing levels (low), type of shift (e.g., on‐call and night shift—Australian studies and rotating roster) and area of practice (e.g., emergency department, intensive care unit and urgent and critical care). Common precipitating factors for occupational violence and aggression in the Australian context are alcohol and drugs, dissatisfaction with service/waiting times and mental health conditions. These findings suggest that triage processes for mental health and alcohol and other drugs are needed to reduce the risk of occupational violence and aggression and may be particularly important when individuals with a history of behaviours of concern present to health services.

### 4.2 RQ 2. What is the *availability* and *effectiveness* of policies / interventions / recommendations that address occupational violence and aggression perpetrated by patients/visitors in rural health service urgent care facilities?

#### 4.2.1 Policies

Hegney et al. (2003) investigated the availability and perceived adequacy of occupational violence and aggression policy among private, public and aged care sector nurses in Australia. Policy content was not examined. In the public sector only, occupational violence and aggression policies were significantly less prevalent in remote areas than in capital cities and large rural centres. Men perceived policies as less effective than did women, and the level of experience was inversely related to perceived effectiveness. Citing differences in occupational violence and aggression across the context of practice, the authors argued that a one‐size‐fits‐all approach is unlikely to be effective. In Lyneham's (2000) study, around 50% of Australian rural and remote nurses reported that their emergency department had a policy relating to occupational violence and aggression. However, (global) satisfaction with administrative responses to occupational violence and aggression varied.

Existing studies fail to elaborate on the content or shortcomings of available occupational violence and aggression policies. Studies of policy effectiveness in rural health settings are needed to inform occupational violence and aggression policy development. These findings are consistent with a recent call for targeted, effectively operationalised legislation, policies and penalties for occupational violence and aggression in non‐metropolitan settings (Hills et al., 2021). Expert consultancy and peer‐review mechanisms for occupational violence and aggression policies, including consultation with best practice forums in other contexts (e.g. mental health and forensics), may advance work in this area.

#### 4.2.2 Interventions and recommendations

##### Pushing the training agenda

With regard to education and training interventions, Fisher et al.' (1996) study identified that almost 50% of Australian remote area nurses had no occupational violence and aggression orientation prior to commencement. Slightly over half received cross‐cultural information, but less than a quarter received personal safety information. Similarly, Lyneham (2000) found that around one‐third of rural emergency nurses and one‐fifth of remote emergency nurses in Australia had never been offered training for occupational violence and aggression. Hills' (2008) study of Australian rural nurses (no drill‐down data for urgent and critical care) found that the median number of times participants had received aggression management training in the previous 5 years was just once. These findings highlight a significant gap in preparation for the prevention and management of occupational violence and aggression in the Australian rural healthcare setting. A literature review on issues concerning Australian remote area nurses by Opie et al. (2011) recommended education and training in aggression management, de‐escalation strategies, behavioural precursors to violence and protective strategies, as well as building positive relations in the community. Luck et al.' (2009) study of Australian regional emergency department nurses servicing a multi‐cultural, rural/remote community identified five broad practices that nurses used to avert or reduce occupational violence and aggression: being safe, available, respectful, supportive and responsive (see Table 1).

With regard to intervention effectiveness, Hills (2008) found that participation in aggression management training in the previous 5 years showed a weak, negative association with the proportion of verbal abuse and a weak, positive association with self‐efficacy. Not surprisingly, 70% of participants reported self‐efficacy for dealing with occupational violence and aggression in the low‐to‐medium range. In the Beattie, Griffiths, et al. (2018a) and Beattie, Innes, et al. (2018b) studies, Australian healthcare workers (urban and regional/remote aggregated) recommended interventions for occupational violence and aggression such as trauma‐informed care and identified emotional intelligence (resilience and mindfulness) and de‐escalation as important training content areas, as opposed to the current focus on managing extreme physical violence, for example, restraint. However, Beattie, Griffiths, et al. (2018a) and Beattie, Innes, et al. (2018b) subsequent studies found that trauma‐informed care practices were not necessarily embedded in education, policies and procedures. Furthermore, in health services that were implementing these practices, data on effectiveness were not available (metropolitan and regional/remote data were aggregated). These findings highlight the need for evidence‐based education and training programmes for occupational violence and aggression, including ongoing training/skills updates. Future research opportunities include a training needs analysis and assessment of self‐efficacy for responding to occupational violence and aggression across different areas of practice.

##### Lockdown and surveillance

Farrell et al.'s (2014) study of Australian midwives and nurses (aggregated across geographic locations, with overrepresentation of rural participants) identified high‐standard patient facilities, personal protective equipment, sufficient staffing levels and effective enforcement of policies by management as protective factors for occupational violence and aggression. Similarly, Opie et al. (2011) recommended the following occupational violence and aggression prevention strategies for remote area nurses: (1) improved security, for example, physical work environment and equipment, and (2) job and task design, for example, ensuring nurses do not work alone. However, the Beattie, Griffiths, et al. (2018a) and Beattie, Innes, et al. (2018b) studies (metropolitan and remote aggregated) found that Australian healthcare workers perceived safeguards (physical and psychological, for example, barriers and signage) as an obstacle to social engagement and trust between workers and consumers, which may exacerbate occupational violence and aggression. Thus, upskilling of healthcare workers in communication skills and trauma‐informed care may be as important as reviewing personal protective equipment, physical work environment features and job/task design.

##### Immediate and ongoing staff support and safety

Australian remote area nurses in Fisher et al.'s (1996) study reported that there was little or no post‐incident support for occupational violence and aggression apart from temporary leave. This may reduce confidence in reporting future incidents; only half of the respondents *always* officially reported incidents. In Lyneham's (2000) study, incident reporting was even lower, and this was attributed to beliefs that violent incidents were ‘part of the job’ and ‘nothing would be done’ by the health service administration. Indeed, more than half of all Australian respondents (rural, remote and metropolitan) in this study reported that they had never received support following serious incidents, with several respondents describing the administration as punitive and blaming staff. Mayhew and Chappell (2003) estimated that only between 8% and 12% of all occupational violence and aggression incidents are formally reported and recorded on official databases (Australian rural and urban data were aggregated across areas of practice). However, updated estimates are needed.

Ross‐Adjie et al. (2007) examined debriefing for stress‐invoking incidents, including occupational violence and aggression, among Australian enrolled nurses (metropolitan and rural aggregated). Almost 60% reported that routine debriefing was not offered. Even when debriefing was available, some staff were unable to attend, as there was no relief from duties. The shift coordinator or most senior nurse on duty often chaired debriefing sessions, but several respondents felt that this should be done independently, to maintain confidentiality. Some respondents reported that debriefing was not helpful/optimal due to chairs being ‘unskilled’. Respondents identified the need for ongoing, rather than one‐off debriefing sessions. Similarly, in Magin et al.'s (2010) study, Australian, rural general practitioner registrars reported that their personal safety concerns were not always taken seriously by their supervisors.

More recent studies are consistent with these findings. Beattie, Griffiths, et al. (2018a) and Beattie, Innes, et al. (2018b) studies (Australian healthcare workers in metropolitan and rural/remote health services) found that debriefing of staff post‐incident was inconsistently offered and frequently delivered by staff with no formal training.

Failure to properly document incidents, debrief and support healthcare workers following occupational violence and aggression is problematic for their psychological well‐being. Left unresolved, past experiences may leave healthcare workers vulnerable to post‐traumatic stress and with reduced capacity to manage future occupational violence and aggression incidents, placing themselves and those in their care at risk (Beattie, Griffiths, et al., 2018a; Beattie, Innes, et al., 2018b). The review by Opie et al. (2011) recommended incident reporting and supporting staff following occupational violence and aggression incidents as essential tertiary strategies for prevention and management. Standard protocols for relief from duties, debriefing and post‐incident support are needed, including immediate and ongoing access to independent psychological support services as required.

Brock et al. (2009) recommended the use of a secure room/security guard system in Australian rural emergency departments to facilitate (1) one‐on‐one observation of patients in a safe environment; (2) additional security, during times when staffing is minimal and (3) early detection of fleeing patients. However, due to the retrospective nature of the study, the authors could not provide quantitative data on the effectiveness of this system in reducing or averting occupational violence and aggression. Nonetheless, the authors urged rural health services to consider constructing a secure room and contracting a security firm. However, not all health services are likely to have access to the funding required to implement such measures.

##### Emergency services presence and linkages

In Lyneham's (2000) study, around one‐fifth of rural and one‐third of remote emergency nurses in Australia reported that there were no security staff at their health services. For health services with security staff, approximately 50% of calls were answered within 10 minutes. Police responded within 30 minutes 84% of the time in rural settings but only 48% of the time in remote settings, reinforcing the notion that geographical distance to a response is a barrier to occupational violence and aggression management. Telephoning police was also a major defence strategy for allied health professionals and nurses in Australian rural health services in Alexander et al.'s (2004) study. Opie et al.'s (2011) review (remote area nurses) recommended developing safety campaigns with a variety of partners, including the police, victim support centres and the media. Outside the context of *rural* health services, Salmon et al. (2021) recommended multi‐agency collaboration as the first step for the prevention and management of occupational violence in hospital settings. They suggested that services need to work collaboratively with emergency services and community organisations to develop local, multi‐agency occupational violence and aggression committees that support whole community responses, including improved strategies for managing occupational violence and aggression while awaiting a police response. This might include collaborating with representatives from ambulance, police, local security firms (where applicable) and other community services. Regular public forums to discuss community safety may also be appropriate to develop local anti‐violence campaigns in health services and the broader community.

## 5 SUMMARY

Fifteen refereed journal articles matched our criteria and were retained for inclusion in the systematic review. With regard to the nature of occupational violence and aggression perpetrated by patients/visitors in rural health service urgent care facilities (RQ1), findings indicated that the most common type of occupational violence and aggression is verbal aggression, with patients as the primary perpetrators. Risk factors are age of practitioner (younger), rurality/remoteness, sector (public), staffing levels (low), type of shift (e.g., on‐call and night shift—Australian studies and rotating roster) and area of practice (e.g., emergency departments, intensive care units and urgent and critical care). Common precipitating factors are alcohol and drugs, dissatisfaction with service/waiting times and mental health conditions.

With regard to the availability and effectiveness of policies / interventions / recommendations that address occupational violence and aggression in rural health services (RQ2), studies failed to elaborate on policy content or shortcomings; thus, it was not possible to address the effectiveness of different policies. Evaluations of education and training interventions were also lacking, but recommendations include de‐escalation skills, trauma‐informed care and resilience training. Physical work environment security and job task design (e.g., staffing levels) are identified as priorities to ensure staff safety. The review also highlighted the importance of an organisational culture that acknowledges and supports staff affected by occupational violence and aggression and a community culture that facilitates collaboration between rural healthcare workers and public sector organisations such as the police.

## 6 STRENGTHS AND LIMITATIONS

This systematic review includes several strengths: pre‐registration, adherence to PRISMA guidelines, clear objectives and narrowly focused research questions and comprehensive database searches with no date restrictions, to allow for the potential inclusion of a broad range of studies. In addition, we conducted a rigorous quality assessment, with a reliable tool, and achieved moderate‐to‐good inter‐rater agreement for quality scores. Finally, we developed and implemented a new index to assess the certainty of the body of evidence, with excellent inter‐rater reliability, thus supporting an objective summary of the literature.

While our review aimed to cover international literature on occupational violence and aggression in rural health service urgent care facilities, we limited our search to literature in the English language due to a lack of resources for translation. Consequently, we might have missed relevant international literature published in other languages. All studies but one was conducted in the Australian context. Additionally, quantitative analysis of the results was not possible due to the heterogeneity of the samples, research methods and data analytic approaches in the included studies. A further limitation was that only some studies reported disaggregated data for the population of interest, which reduces the generalisability of the findings to the target population of rural healthcare workers in urgent and critical care.

The systematic review is also limited by the quality of the included studies, with fewer than 50% of the studies being of high quality. The most common study limitations included sample representativeness relative to the target population (quantitative); absence of clear links among data sources, collection and analysis and interpretation (qualitative) and insufficient explanation of divergences/inconsistencies between quantitative and qualitative results and overall quality, based on adherence to each tradition (mixed methods studies).

With regard to certainty, most studies performed well on relevance to the research questions, but the relevance of the sample was often compromised (see above). This made it difficult to fully differentiate occupational violence and aggression type, frequency, severity, perpetrator characteristics and risk factors in rural versus metropolitan health services. Further research is needed to establish the nature and prevalence of occupational violence and aggression perpetrated by patients/visitors in rural health service urgent care settings, the effectiveness of relevant policies/interventions/recommendations in rural health settings and related health and safety outcomes.

Finally, it should be noted that this systematic review was undertaken before COVID‐19, and it is possible that the pandemic may have reduced, exacerbated or altered the presentation of occupational violence and aggression in health services. The study may provide a baseline for comparison of subsequent studies of occupational violence and aggression in rural health services in the context of the pandemic.

While the present study was under review, studies that might have met the criteria for inclusion in this review could have been subsequently published. Thus, it is important to acknowledge that this review may not include all relevant studies published around the time the review was completed.

## 7 CONCLUSION

Based on the findings of the review, research recommendations include systematic identification and evaluation of occupational violence and aggression policies and education and training interventions in rural health services settings. Such information is not currently available in the literature.

Clinical recommendations concern balancing security upgrades with trauma‐informed care for patients in distress. Consistent changes to the physical work environment and job/task design in rural health services should be made as a priority to ensure staff safety.

Policy recommendations include organisational policies that support an anti‐violence culture by encouraging incident reporting, debriefing (by independent and suitably qualified individuals) and ongoing post‐incident support to promote staff well‐being. The literature reinforces the importance of collaboration between rural health services and police and community organisations to support healthcare workers' responses to occupational violence and aggression.

## AUTHOR CONTRIBUTIONS

Sharon Grant: 45% set the parameters for the review, completed article screening, completed data extraction, completed quality appraisal and certainty ratings and manuscript writing (lead). Stephanie Hartanto: 45% set the parameters for the review, completed PROSPERO registration, completed database searches and upload to Covidence, completed article screening, completed quality appraisal and certainty ratings, manuscript writing and formatting to template. Diane Sivasubramaniam: 5% Assisted with screening, that is, resolving disagreements with article inclusion/exclusion and quality appraisal ratings and manuscript editing. Kaye Heritage: 5% Assisted with setting the parameters for the review and provided feedback on data extraction and thematic analysis and manuscript editing.

## FUNDING INFORMATION

Co‐funded by Swinburne Social Innovation Research Institute Interdisciplinary Seed Funding Scheme and SMART Rural Health Network.

## CONFLICT OF INTEREST

On behalf of all authors, the corresponding author states that there is no conflict of interest.

## PROSPERO LINK

http://www.crd.york.ac.uk/PROSPERO/display_record.php?ID=CRD42019131867

## Supporting information


Data S1

## Data Availability

Data sets not applicable to this article as no data sets were generated or analysed during the current study.
